# Porous media properties of reticulated shape memory polymer foams and mock embolic coils for aneurysm treatment

**DOI:** 10.1186/1475-925X-12-103

**Published:** 2013-10-12

**Authors:** Andrea D Muschenborn, Jason M Ortega, Jason M Szafron, David J Szafron, Duncan J Maitland

**Affiliations:** 1 Texas A&M University, 3120 TAMU, 77843 College Station, Texas, USA; 2 Lawrence Livermore National Laboratory, 7000 East Ave., L-090, 94551 Livermore, CA, USA

**Keywords:** Permeability, Form factor, SMP, Shape memory polymer foams, Embolic coils, FHDD, Forchheimer-Hazen-Dupuit-Darcy equation, Aneurysm treatment

## Abstract

**Background:**

Shape memory polymer (SMP) foams are being investigated as an alternative aneurysm treatment method to embolic coils. The goal of both techniques is the reduction of blood flow into the aneurysm and the subsequent formation of a stable thrombus, which prevents future aneurysm rupture. The purpose of this study is to experimentally determine the parameters, permeability and form factor, which are related to the flow resistance imposed by both media when subjected to a pressure gradient.

**Methods:**

The porous media properties—permeability and form factor—of SMP foams and mock embolic coils (MECs) were measured with a pressure gradient method by means of an *in vitro* closed flow loop. We implemented the Forchheimer-Hazen-Dupuit-Darcy equation to calculate these properties. Mechanically-reticulated SMP foams were fabricated with average cell sizes of 0.7E-3 and 1.1E-3 m, while the MECs were arranged with volumetric packing densities of 11-28%.

**Results:**

The permeability of the SMP foams was an order of magnitude lower than that of the MECs. The form factor differed by up to two orders of magnitude and was higher for the SMP foams in all cases. The maximum flow rate of all samples tested was within the inertial laminar flow regime, with Reynolds numbers ranging between 1 and 35.

**Conclusions:**

The SMP foams impose a greater resistance to fluid flow compared to MECs, which is a result of increased viscous and inertial losses. These results suggest that aneurysms treated with SMP foam will have flow conditions more favorable for blood stasis than those treated with embolic coils having packing densities ≤ 28%.

## Introduction

Three to six million people are estimated to have an intracranial saccular aneurysm (ISA) in the United States [1]. While the potential of an ISA rupture is relatively low (0.1-1%), 35-50% of the patients who do experience an ISA rupture die [2]. From those who survive, only 50% regain functional independence, while 30% are permanently disabled and 20% require institutional care [3,4]. Among the common risk factors that are found in the different theories for the initiation of ISAs are the geometry of the cerebral vasculature, genetics, hemodynamics, hypertension, smoking, and gender (65% incidence in women) [4-7].

The most common endovascular approach to treat ISAs is the insertion of embolic coils into the aneurysm sac to cause the formation of a stable thrombus, thereby isolating the aneurysm from the vasculature and preventing its future rupture [4,8-10]. Despite the thousands of patients who have been successfully treated with embolic coils, there are still considerable limitations to this treatment technique. These include incomplete filling of the aneurysm resulting in low coil packing densities, coil compaction over time, and coil migration to the parent artery [11-13]. For example in aneurysms with a diameter ≥ 6E-3 m, the maximum packing density is on average only 21% [14].

To overcome these limitations, polyurethane shape memory polymer (SMP) foams have been proposed as an alternative aneurysm treatment [15-20]. By elevating the temperature of the SMP foam above its glass transition temperature (T_g_), it can be deformed into a metastable secondary geometry, which is retained upon subsequent cooling below T_g_[16,21,22]. Increasing the temperature above T_g_ subsequently results in recovery of the primary shape. The main advantage of this treatment technique is that the SMP foams show up to 70 times volume expansion [16]. This feature enables them to be delivered through a microcatheter and to completely fill an aneurysm upon actuation. By mechanically-reticulating the SMP foam, *i.e.*, piercing it with a needle or wire, the hemodynamics within the treated aneurysm can be further customized.

In order to evaluate how such hemodynamics can affect thrombus formation, several previous studies have utilized a porous media approach for both foam- and coil-filled aneurysms [23-27]. The majority of these studies have been performed computationally. For example, Kakalis et al. and Mitsos et al. employed the Kozeny theory to calculate the permeability of embolic coils using the porosity and specific surface, which is the total interstitial surface area per unit bulk volume of the porous medium [23,26]. In the present study, however, an experimental approach was employed to measure and compare the permeability and form factor of SMP foams and mock embolic coils (MECs), which are inexpensive, non-clinical coils that approximate the diameter, distribution, and packing density of clinical embolic coils. In this study, we utilized the Forchheimer-Hazen-Dupuit-Darcy (FHDD) equation:

(1) $$-\frac{\partial P}{\partial x}=\frac{\mu }{K}v_{0}+\mathrm{\rho C}v_{0}^{2}$$

where $\frac{\partial P}{\partial x}$ is the pressure gradient along the sample in the direction of flow (Pa/m), *μ* is the dynamic viscosity of the fluid (Pa · s), *K* is the intrinsic permeability of the sample (m^2^), *v*_*0*_ is the Darcy velocity (flow rate, Q, divided by cross-sectional area of the sample) (m/s), *ρ* is the density of the fluid (kg/m^3^), and *C* is the form factor of the sample (m^-1^). The permeability and form factor are constitutive geometrical parameters of the porous matrix that depend upon the contributions of viscous drag and inertial losses, respectively [28-30]. Thus, the permeability is inversely proportional to the surface area of contact between the porous matrix and the fluid and the form factor is proportional to the projected cross-sectional area of the obstructing matrix perpendicular to the flow direction. This equation is generally applicable for any type of porous media where viscous-dominated flow at low velocities transitions to inertia-dominated flow [31]. In cases when flow velocities are low, *i.e.*, usually at Reynolds numbers less than 1, this equation reduces to the Darcy equation [32]. In the present study, we compared the permeability and form factor of mechanically-reticulated SMP foams to those of MECs. Strictly from a fluid dynamics perspective, the purpose of this work is to evaluate the potential of SMP foams as embolic devices or components of embolic devices.

## Materials and methods

### Flow system and measuring apparatus

**Figure 1:**
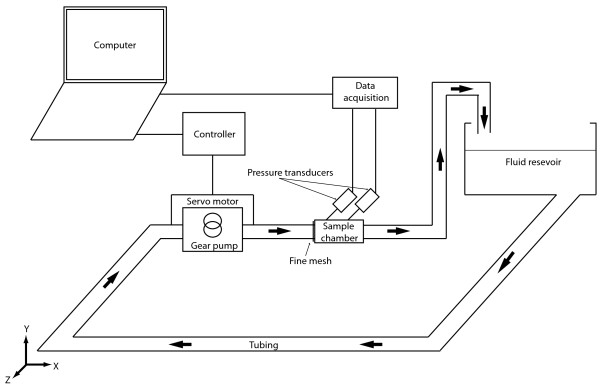
**Flow system constructed to measure the pressure gradient along the SMP foam and MEC samples.** The pressure gradient was measured utilizing two high accuracy pressure transducers, and the flow rate was measured by hand with a graduated cylinder and a digital stop watch. A fine mesh (stainless steel 316, 0.1E-3 m wire diameter, 4E-7 m^2^ squares) was installed at the inlet of each sample chamber to homogenize the incoming flow.

An *in vitro* closed flow loop was constructed to produce a measurable pressure gradient across either the SMP foam or the MEC sample at a given flow rate (see Figure 1). The flow loop was comprised of a gear pump (Chemsteel R106, Oberdorfer), a servo motor (750 W M-series, Applied Motion Products), a motor controller (BLuAC5-Q, Applied Motion Products), a testing chamber, a fluid reservoir of room temperature water elevated 0.3 m above the testing chamber, and two high accuracy pressure transducers (PX429-2.5G5V, Omegadyne Inc. with an accuracy rated at ± 0.08% of the baseline maximum). The output voltage of the pressure transducers was recorded at 1 Hz through a data acquisition system (USB6251, National Instruments) to a computer disk for 120 seconds. The flow rate was measured by hand using a digital stop watch (Traceable, VWR) and a graduated cylinder (250 ml capacity) to within an uncertainty of 4%.

**Figure 2:**
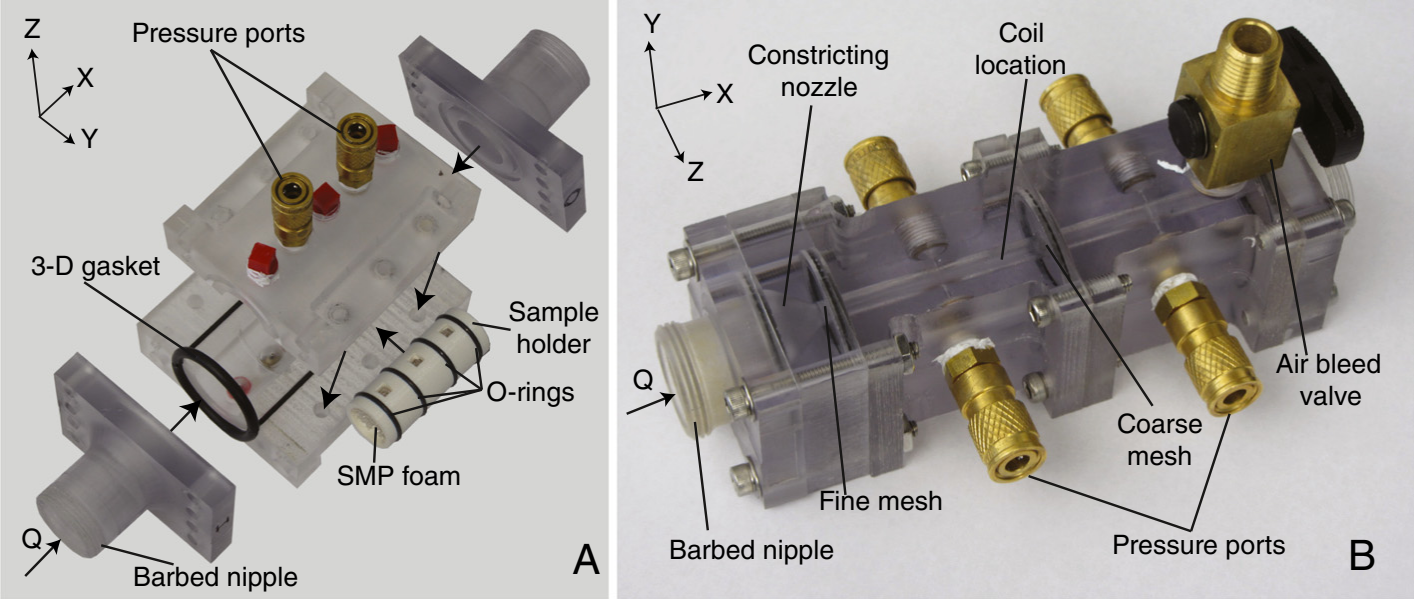
**Exploded view of the SMP foam chamber (A) and assembled MEC chamber (B).** The arrows in **(A)** indicate how the components were assembled. A 3-D gasket was fabricated and used to ensure the SMP foam chamber was water tight, and O-ring grooves were machined in the lumen of the chamber to aid in the repeatable alignment of the sample holders and sealing of the pressure ports. For the MEC chamber, a constricting nozzle was implemented in its design to provide a smooth transition from the 20E-3 m diameter tubing to the 6E-3 m diameter of the chamber lumen. A coarse mesh (stainless steel 316, 0.25E-3 m wire diameter, 2.3E-6 m^2^ squares) was installed downstream of the packed MECs to hold them in place during the testing procedure.

Two distinct chambers were required for the characterization of both media. The chamber for the SMP foam samples was fabricated from polycarbonate (inlet and outlet) and poly(methyl methacrylate) (body) using a computerized numerically controlled (CNC) milling machine (MDX540, Roland). As pictured in Figure 2(A), the body of the chamber was designed to enclose a sample holder (inner diameter and outer diameter 15.1E-3 m and 18.9E-3 m, respectively), to which SMP foam samples were adhered prior to testing. The dimensions of the SMP foam samples were chosen such that they were large enough to minimize any effects of heterogeneities in their structure. Using a 3-D printer (Fortus 360, Stratasys), the sample holders were fabricated with three 4E-3 × 4E-3 m pressure port openings, 15E-3 m apart of one another. Only the upstream and downstream pressure ports were utilized in the measurements. O-ring grooves were machined in both the chamber body and the sample holder to ensure repeatable chamber alignment and to seal the pressure ports. A fine mesh (stainless steel 316, 0.1E-3 m wire diameter, 4E-7 m^2^ squares) was installed at the inlet of the chamber to homogenize the incoming flow.

The chamber for measuring the MECs was fabricated from polycarbonate using the Roland CNC (Figure 2(B)). A constricting nozzle was machined at the inlet to provide a smooth transition from the 20E-3 m diameter tubing to the 6E-3 m diameter of the chamber lumen, which is within the range of diameters for ISAs [14]. A fine mesh (stainless steel 316, 0.1E-3 m wire diameter, 4E-7 m^2^ squares) was installed downstream of the nozzle to homogenize the incoming flow. Two pressure ports 50E-3 m apart were utilized to connect the pressure transducers. A coarse mesh (stainless steel 316, 0.25E-3 m wire diameter, 2.3E-6 m^2^ squares) was installed between the downstream edge of the coils and the second pressure transducer in order to hold the MECs in place during the testing procedure. The pressure gradient across this mesh was subtracted from the pressure gradient data for each MEC sample.

### Sample preparation

**Figure 3:**
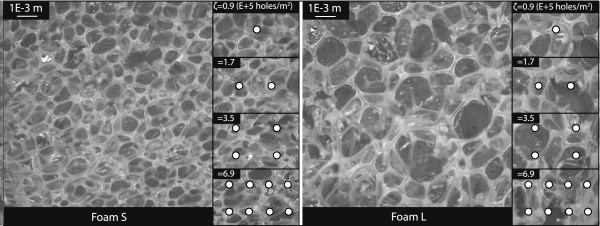
**SMP foams prior to mechanical reticulation and mechanical reticulation scenarios (2.5× magnification).** The solid white circles represent the holes due to mechanical reticulation (to scale). Hole density (ζ) was calculated by dividing the number of holes pierced by the surface area of the 3-D-printed template. The average cell size of foams S and L was 0.7E-3 and 1.1E-3 m, respectively.

Two SMP foams with different cell sizes were evaluated in this study. They were labeled as foam S, for small average cell size, and L, for large average cell size (see Figure 3). The SMP foams were made by a combined physical and chemical blowing process and cleaned through a series of washes utilizing 0.1 N hydrochloric acid and Contrad soap solution with sonication (the chemistry of the SMP foams can be found in [21]). Different cell sizes were achieved by varying the viscosity of the pre-polymer mix. Foam S had a T_g_ measured by differential scanning calorimetry (DSC) of 59.1°C, an average cell size of 0.7E-3 m, and a porosity of 98%. Likewise, Foam L had a T_g_ of 55.6°C, an average cell size of 1.1E-3 m, and a porosity of 98% (refer to [16] for the porosity calculation). The SMP foam samples were cut from a large piece of dried SMP foam using a drill press and a 1.905E-2 m (¾”) hole saw attachment. The appropriate axial length was then cut using a straight-edge razor. The resulting SMP foam samples had a diameter of ~16E-3 m and a length of 49E-3 m. For mechanical reticulation, the SMP foam samples were perforated utilizing a stainless steel acupuncture needle (Kingli) with a diameter of 0.3E-3 m. In order to achieve mechanical reticulation in a repeatable manner, we 3-D printed a hollow cylinder with a cap and utilized it as a template guide. The hollow cylinder had 1600 radial holes and 124 axial holes with a hole diameter of 0.9E-3 m and was designed so that the SMP foam samples fit securely within it during reticulation. Four scenarios of mechanical reticulation were produced, and they are reported as a function of hole density (ζ), *i.e.,* the number of holes pierced per unit surface area of the SMP foam sample. The resulting hole densities were 0.9, 1.7, 3.5 and 6.9E + 5 holes/m^2^. In all four cases, the pierced holes were evenly distributed along the surface of the SMP foam sample. Figure 3 illustrates a true-scale representation of the four hole densities overlaid on each foam type. Following mechanical reticulation, the SMP foam samples were fixed to the sample holders by being crimped to a diameter of roughly 8E-3 m and then allowed to expand inside the sample holder, which had been evenly coated with a thin layer of water resistant epoxy (Marine Epoxy, Loctite). By allowing the foams to actuate and conform to the lumen of the sample holders, we replicated what would happen *in vivo* as the foams would conform to patient-specific geometries. The epoxy was allowed to cure for at least 2 hours prior to beginning each test. Since long term exposure to water can potentially affect the mechanical properties of the SMP foams, data were acquired at 0, 2, and 4 hours of water exposure times [16,18].

**Figure 4:**
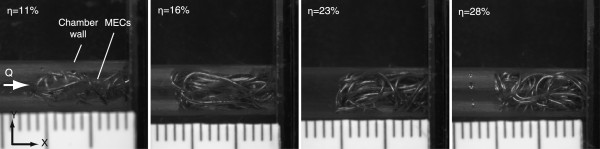
**Packed MECs in the measuring chamber. Coil lengths of 0.28, 0.47, 0.59 and 0.71 m were randomly packed to achieve packing densities of 11, 16, 23 and 28%, respectively.** Packing density (η) was calculated by dividing the volume of the coil by the cylindrical bounding volume of the coil mass.

MEC samples were prepared by randomly coiling different lengths (*i.e.,* 0.28, 0.47, 0.59 and 0.71 m) of copper wire with a diameter of 0.39E-3 m, which is near the center of the diameter range for commercially-available embolic coils [9]. Each MEC sample was then packed inside the measuring chamber as shown in Figure 4. The packing density (η) was calculated as the ratio of the volume of the coil to the cylindrical bounding volume of the coil mass. The resulting packing densities were 11, 16, 23 and 28%.

### Sample characterization

For each sample, a second-order least squares fit to the pressure gradient versus Darcy velocity was implemented. The values of the permeability, *K*, and form factor, *C*, were then calculated using Equation 1 [33]. A total of nine values of *v*_*0*_ and their corresponding pressure gradients were utilized for the calculations. Measurements were made to check for hysteresis, which could have been caused by sample deformation during testing [30]. To quantify other potential sources of measurement variability arising from the instrumentation, an uncertainty analysis was performed based upon the root-sum-squares method [34].

The Reynolds number *Re* of these measurements was defined as the ratio of inertial forces to viscous forces (from Equation 1) [28,35]

(2) Re=ρυoKCμ

Non-dimensionalization of Equation 1 by *Cρυ*_0_^2^ yielded

(3) −∂P∂xCρυ02=f=1Re+1

where *f* is the friction factor [35,36].

## Results

**Figure 5:**
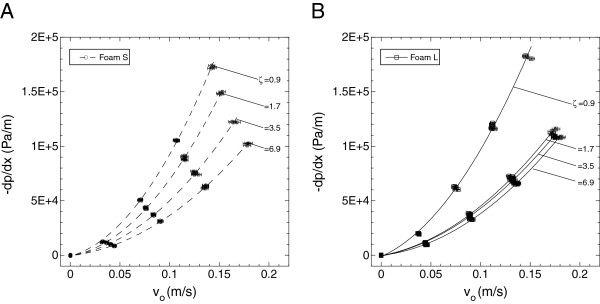
**Pressure gradient versus Darcy velocity of reticulated SMP foams S (A) and L (B).** The mechanical reticulation hole density (ζ) has units E + 5 holes/m^2^. As the hole density increases, the pressure gradient across the samples also decreases for both SMP foam types.

**Figure 6:**
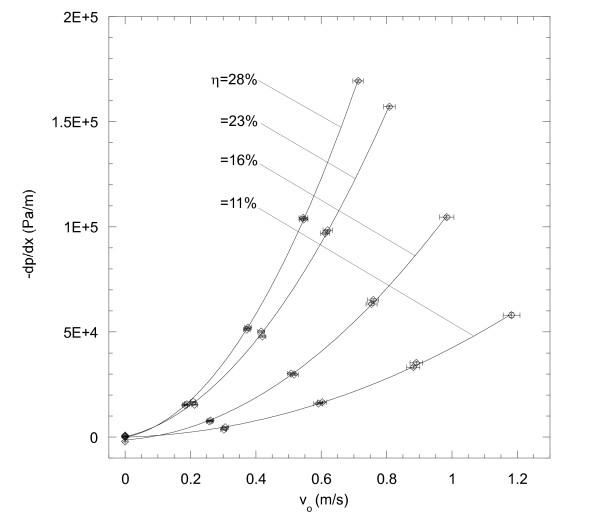
**Pressure gradient versus Darcy velocity of MECs for various packing densities, η.** By increasing the packing density of the MECs, the pressure gradient across the samples also increases.

Plots of the pressure gradient versus Darcy velocity are shown in Figures 5 and 6 for the SMP foams and MECs, respectively. There was no evidence of compression-induced hysteresis for either media. Additionally, moisture uptake from water exposure of the SMP foams had little, if any, impact on the measured quantities. Therefore, data from all water exposure times were used in the least squares fits for the SMP foams. For all the regressions, the coefficients of determination for both media were greater than R^2^ = 0.99.

**Figure 7:**
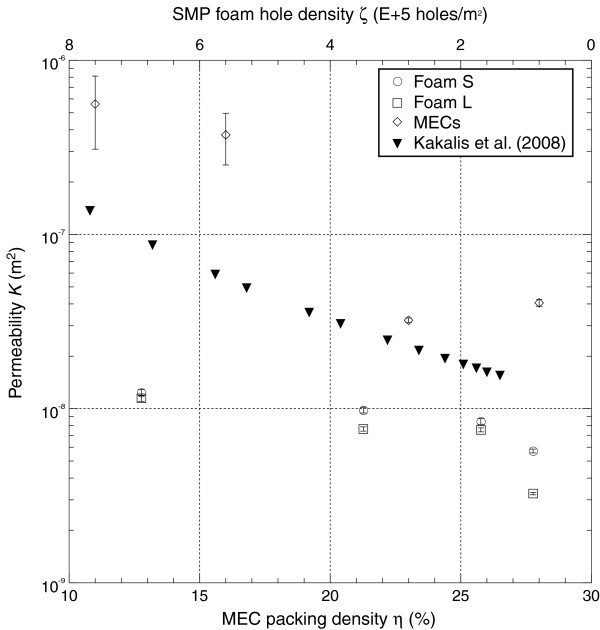
**Permeability of the SMP foams and MECs compared to that calculated for embolic coils **[23]**.** The permeability of the MECs was higher than the permeability of both SMP foam types in all cases. Lowering the packing density resulted in an increase in permeability, while decreasing the hole density resulted in a decrease in permeability. The permeability data for embolic coils that was calculated using the Kozeny theory by Kakalis et al. [23] follows a similar trend as our measured MEC data.

**Figure 8:**
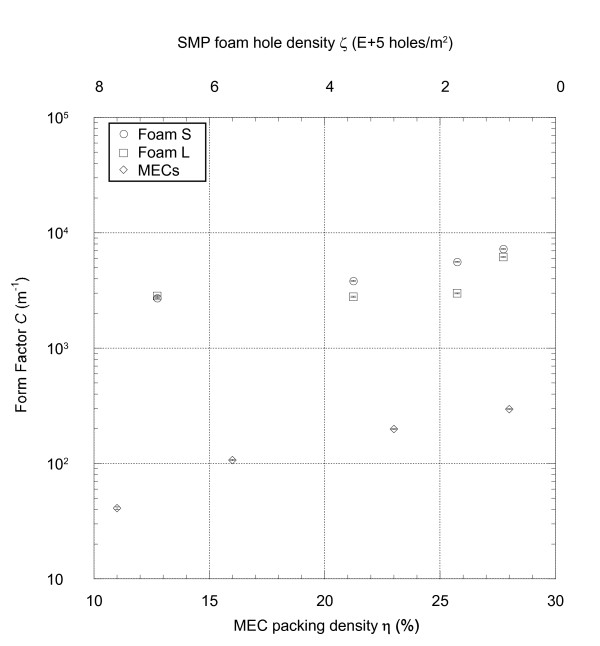
**Form factor of the SMP foams and MECs.** The form factor of both SMP foam types was higher than that of the MECs in all cases, and it increased for SMP foam samples with lower hole densities. The form factor of the MECs was higher for samples with higher packing density.

Plots of the permeability and form factor are shown in Figures 7 and 8, respectively. The differences in the permeability and form factor for the SMP foams due to varying the average cell size were not as pronounced as those resulting from varying the amount of mechanical reticulation. As would be expected, higher amounts of mechanical reticulation resulted in overall higher permeability and lower form factor values. For foam S, the permeability values ranged between 5.70E-9 and 12.4E-9 m^2^_,_ and the form factor values ranged between 2.72E + 3 and 7.26 + 3 m^-1^. For foam L, permeability and form factor values ranged from 3.25E-9 to 11.5E-9 m^2^ and 2.80E + 3 to 6.19 + 3 m^-1^ respectively. For the MECs, there was an order of magnitude change in the permeability and form factor for samples with η = 11% versus samples with η = 28%; the permeability and form factor ranged from 40.5E-9 to 561E-9 m^2^ and 0.0409E + 3 to 0.296E + 3 m^-1^ respectively.

**Figure 9:**
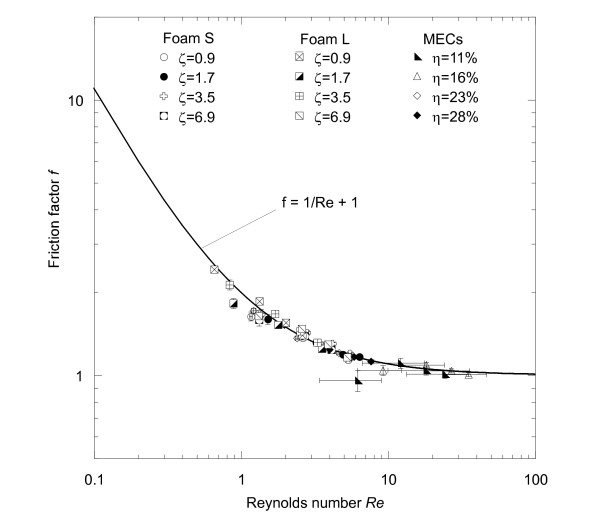
**Friction factor versus Reynolds number for the SMP foam samples and MECs.** The mechanical reticulation hole density (ζ) has units E + 5 holes/m^2^. The friction factors and Reynolds numbers for both SMP foam types and for the MECs collapse onto the relationship specified by Equation 3 for Reynolds numbers ranging approximately from 1 to 35. The friction factors of all SMP foam samples were higher than those of the MECs.

Figure 7 depicts additional permeability data for embolic coils (diameter of 0.25E-3 m) that were considered in the simulations of Kakalis et al. (2008) [23]. Their calculated permeability values follow a similar trend to that of the MECs, but are on average four times smaller. When the measured permeability and form factors are used to non-dimensionalize the pressure gradient and Darcy velocity, the resulting friction factors and Reynolds numbers for both SMP foams and MECs collapse onto the relationship specified by Equation 3 for Reynolds numbers ranging from approximately 1 to 35 (Figure 9).

## Discussion

The goal of state-of-the-art aneurysm treatments is to reduce blood flow into the aneurysm by filling it with embolic coils that cause the aneurysm to acutely fill with thrombus, thereby preventing future rupture. This thrombus formation process arises not only from biochemical reactions, but also from altered hemodynamics, such as flow stagnation [4,8,9,37]. The current study demonstrates that SMP foams impose a greater resistance to flow compared to MECs and would therefore be more effective at producing these desired post-treatment hemodynamics. We do not observe hysteresis in the pressure gradient versus flow speed data, an effect which would be indicative of sample deformation during the test [32]. Therefore, the higher friction factors for the SMP foams arise from a larger contact surface area and projected area that produce greater viscous and inertial losses, respectively, relative to those of MECs. These results consider the scenario of foam packing the aneurysm at a 1:1 volume ratio, not including the ability to oversize the foam, which has been proven to be safe for foams sized up to twice the size of an aneurysm [38]. Such oversizing would result in even greater stagnation within the treated aneurysm.

The porous media theory described in this study has been previously used to measure the geometrical parameters of polyurethane foams and fibrous porous media [39-42]. Additionally, blood flow through aneurysms treated with either embolic coils [23-26] or SMP foams [27] has been simulated utilizing porous media models. Major differences between these various studies are the selection of the porous media model, the definition of the Reynolds number, the flow regime of the experiments, and the fluid type. Some studies limit the flow range to the Darcy regime and define the Reynolds number based upon the cell size [31,43], while others use different porous media models, such as the Kozeny theory [23]. The greatest challenge of this type of model is that it requires the characterization of additional constitutive parameters, such as the specific surface and, in some cases, the flow path tortuosity [23,26,41]. Another drawback specific to the Kozeny theory is that it does not take into account the diverging or converging nature of the flow within the porous medium. This is one possible reason for the differences between the embolic coil permeability values of Kakalis et al. and those measured for MECs [23,32].

One of the main limitations in this study is the selection of the Newtonian working fluid, which does not exhibit the shear thinning behavior of blood at small shear rates [44]. Even though it is well documented that the permeability and form factor are intrinsic geometrical parameters independent of the working fluid, future experiments must be performed to apply the results of the present study to non-Newtonian blood flow [28,30,32]. Nonetheless, Sorteberg et al. (2004) observed marked similarities in the pressure and fluid dynamics within a bench-top aneurysm model packed with clinical embolic coils using either blood or a saline solution [45]. Furthermore, Ortega et al. (2013) found nearly identical permeability and form factor values for both Newtonian and non-Newtonian viscosity models when simulating blood flow through virtually-reticulated SMP foam at Reynolds numbers (Equation 2) between 0.2 and 2.7 [27]. Thus, within this range of Reynolds numbers, the permeability and form factor values from the present study may be applicable for non-Newtonian blood flow within foam- or coiled-filled aneurysms.

## Conclusions

We have reported *in vitro* experimental measurements on the porous media properties of mechanically-reticulated SMP foams and MECs. For the reticulation and coil packing densities evaluated in this study, SMP foams impose a larger flow resistance than MECs. From a perspective in which only the fluid dynamics are considered, these results suggest that SMP foams will be more effective than embolic coils at inducing flow stagnation within a treated aneurysm.

## Nomenclature

$$\frac{\partial P}{\partial x}$$

Pressure gradient along the porous medium (Pa/m)

*μ* Dynamic viscosity of the working fluid (Pa · s)

*K* Permeability (m^2^)

*v*_*0*_ Darcy velocity (=volumetric flow rate/cross-sectional area of sample) (m/s)

*ρ* Density of the working fluid (kg/m^3^)

*C* Form factor (m^-1^)

*Q* Volumetric flow rate (m^3^/s)

*Re* Reynolds number

*f* Friction factor

ζ Post-mechanical reticulation hole density of the SMP foams (E + 5 holes/m^2^)

η Mock embolic coil packing density (%)

## Abbreviations

SMP: Shape memory polymer; MEC: Mock embolic coil; ISA: Intracranial saccular aneurysm; DSC: Differential scanning calorimetry.

## Competing interests

The authors declare that they have no competing interests.

## Authors’ contributions

ADM: participated in the conceptualization of this study, as well as the design of the experimental setup. She fabricated and assembled the experimental setup, prepared the samples, acquired the data, analyzed and interpreted the data, produced the figures and wrote the manuscript. JMO: participated in the conceptualization of this study, the design of the experimental setup, the analysis and interpretation the data, and the manuscript editing. JMS: contributed significantly in the sample preparation, data acquisition, data analysis and interpretation. He also helped in the figure production and manuscript editions. DJS: helped with the fabrication, assembly and preliminary testing of the experimental setup, as well as with coding for data acquisition and analysis. DJM: participated in the conceptualization of this study, analysis of the data, the manuscript editing and had final approval of the submitted manuscript. All authors read and approved the final manuscript.
